# Breast cancer preclinical models: a vital resource for comprehending disease mechanisms and therapeutic development

**DOI:** 10.17179/excli2024-7973

**Published:** 2025-02-19

**Authors:** Ravneet Kaur, Anuradha Sharma, Nalaka Wijekoon

**Affiliations:** 1 Department of Molecular Biology and Genetic Engineering, School of Bioengineering and Biosciences, Lovely Professional University, Punjab-144411, India; 2 Interdisciplinary Center for Innovation in Biotechnology and Neuroscience, Faculty of Medical Sciences, University of Sri Jayewardenepura, Nugegoda, 10250, Sri Lanka; 3 Department of Cellular and Translational Neuroscience, School for Mental Health and Neuroscience, Faculty of Health, Medicine & Life Sciences, Maastricht University, 6200, Maastricht, The Netherlands

**Keywords:** breast cancer, breast cancer types, preclinical model, women health, triple negative breast cancer

## Abstract

A significant obstacle in translating innovative breast cancer treatments from bench to bed side is demonstrating efficacy in preclinical settings prior to clinical trials, as the heterogeneity of breast cancer can be challenging to replicate in the laboratory. A significant number of potential medicines have not progressed to clinical trials because preclinical models inadequately replicate the complexities of the varied tumor microenvironment. Consequently, the variety of breast cancer models is extensive, and the selection of a model frequently depends on the specific inquiry presented. This review aims to present an overview of the existing breast cancer models, highlighting their advantages, limitations, and challenges in the context of innovative drug discovery, thereby offering insights that may be advantageous to future translational studies. Conventional monolayer cultures are critical for elucidating the different breast cancer types and their behavior, have limitations in adequately replicating tumor environments. The 3D models such as patient-derived xenografts, cell-derived xenografts and genetically engineered models offer better insights by maintaining tumor microenvironments and cellular heterogeneity. Results can be further enhanced when compared with breast epithelial cells, a negative control to determine early stages by investigating differences between healthy and cancerous mammary cells. While cell lines such as MCF-7, MDA-MB-231 etc are useful *in vitro* models, they exhibit genetic variations that may affect drug responses over time. Additionally, animal models, particularly rodents, are instrumental in breast cancer research due to their biological resemblances to humans and the relative ease of genetic modification, however, witness a low occurrence of tumors. This review thus concludes that different preclinical models have their associated benefits and pitfalls. Therefore, specific preclinical models can be created by altering the gene expression at the genetic level or could be selected as per specific experimental needs which will enable successful translation of preclinical findings into clinical trials can be possible.

See also the graphical abstract(Fig. 1).

## List of Abbreviations Used

BC: Breast Cancer

CDX: Cell-Derived Xenografts

ER: Estrogen

GEMM: Genetically Engineered Mouse Model

Her2: Human Epidermal Receptor 2

NHP: Non-Human Primates

NIH: National Institute of Health

PDX: Patient-Derived Xenograft

PR: Progesterone

TNBC: Triple Negative Breast Cancer

WHO: World Health Organisation

## Introduction

Biologically, breast cancer is the uncontrolled proliferation of cells in any part of the breast leading to a highly complex tumor formation. It is characterized by the formation of a lump, which is an extra mass of cells or tissues. These lumps may be felt as a hard mass bulging out or within the skin, the presence of a lump may or may not be accompanied by pain, swellings, or redness. Both men and women are susceptible to developing breast cancer, however, it is more frequent in females. It has significant socioeconomic repercussions and is the second most frequently diagnosed malignancy in women, after skin cancer (Omene and Tiersten, 2010[51]).

The WHO reports that breast cancer is a prevalent malignancy, with over 2.3 million cases reported annually. It is the second leading cause of cancer-related mortality among women worldwide. Nonetheless, a notable discrepancy exists in breast cancer survival rates both among and within various countries. According to the WHO in 2020, 2.3 million females were diagnosed with breast cancer, resulting in a total of 685,000 fatalities, while 7.8 million women were alive who had received a diagnosis within the preceding five years (WHO, 2024[77]). Due to population expansion and aging, the incidence of breast cancer is projected to increase to over 3 million new cases and 1 million fatalities yearly by 2040 (Arnold et al., 2022[2]). 

Being a multifactorial disease, the causative factors for breast cancer are diverse. It develops in response to many biological and molecular changes in the body, such as environmental, genetic, and hormonal changes. 5-10 % of cases of breast cancer are suggested to be inherited, and the onset of the remaining 90-95 % of breast cancers is suggested due to environmental and lifestyle factors (Kolak et al., 2017[31]). The pathology of breast cancer is also very complicated with multiple types based on the tissues involved, molecular mechanisms, and genes involved as well as based on aggressiveness. Breast cancer (BC) can be divided into three major classes based on genetic and histological evidence: - Breast cancer displaying hormone receptors (estrogen receptor (ER+)) or progesterone receptor (PR+), - Breast cancer exhibiting human epidermal receptor 2 (HER2+), and - triple-negative breast cancer (TNBC).

Breast malignancy-exhibiting hormones include cancers that show estrogen (ER+), progesterone (PR+), or human epidermal receptor-2. A very aggressive phenotype with a dismal prognosis is triple-negative breast cancer (TNBC) which is characterized by the absence of all three receptors. Additionally, using DNA expression profiles and ontological studies, Lehmann et al. discovered six types of Triple-Negative Breast Cancer subcategories (TNBCtype-6 categorization) in 2011 (Lehmann et al., 2011[33]). These subcategories include luminal androgen receptor (LAR), immunomodulatory (IM), mesenchymal (M), mesenchymal stem cell-like (MSL), and basal-like 1 (BL-1) and basal-like 2 (BL-2) (Barzaman et al., 2020[5]). 

Despite the application of several treatments and therapies, including hormone therapy, mastectomy, and radiation, factors such as tumor heterogeneity and advanced stage pose challenges that render existing procedures ineffectual. Advancements in screening methodologies, diagnostic practices, and therapeutic interventions have led to a notable enhancement in the overall survival rate of breast cancer patients throughout time. Nonetheless, numerous gaps remain in comprehending the origins, development, and progression mechanisms of different types of breast cancers. Understanding the molecular characteristics of various breast cancer types is essential for the advancement of innovative treatment approaches (Nounou et al., 2015[49]). We use different pre-clinical models to mimic the different types of breast cancer. These lacunae could be responsible for undesired results or major drug failures at clinical levels/ trials even after extensive research at *in vitro* and *in vivo* levels. For example, despite recent improvements in nanomedicine development for BC treatment, cancer nanomedicines frequently fail at the clinical trial stages after successfully passing all the preclinical phases. These factors suggest the need to review about our existing pre-clinical models and fill the research lacunae affecting therapeutic development and efficacy. There could be multiple reasons associated with drug failure at the clinical level despite preclinical efficacy e.g. the stromal population is not represented in standard *in vitro* models, no or pseudo three-dimensional (3D) structure, resulting in poor portrayal of inter- and intra-tumor heterogeneity (Boix-Montesinos et al., 2021[7]). The absence of experimental model systems that adequately depict the diverse manifestations of this disease constitutes a substantial barrier to enhancing our comprehension of breast cancer biology. Owing to the intricacy and diversity of this disease, no singular preclinical model can be anticipated to faithfully depict this cancer type (Vargo-Gogola and Rosen, 2007[70]). 

Thus, development of accurate pre-clinical models that replicate tumor characteristics is required for the effective clinical translation of anticancer to assess their safety and efficacy and more critically, to find biomarkers for therapeutic response. Although commonly used traditional two-dimensional (2D) cell models of cancer have made major contributions to translational research in the field of cancer medicine. These have limited translational efficiency since they show considerable variances from the actual illness. 

The lack of cellular heterogeneity and the tumor microenvironment (TME), which significantly influence cancer progression and treatment resistance, represent the primary limitations of 2D cancer cell models. Advanced models that effectively represent the intricacies and fluctuations of human disease should promote the development of relevant treatment strategies that will significantly improve patient outcomes (Boix-Montesinos et al., 2021[7]). The intricate interactions between complex multicellular structures and the cell-extracellular matrix that contribute to the onset and progression of breast cancer cannot be entirely replicated in cell culture settings. Therefore, utilizing xenografts of cell lines and clinical isolates of breast cancer allows for the investigation of human breast cancer cells within the *in vivo* context (Vargo-Gogola and Rosen, 2007[70]).

## Pre-Clinical Models for Studying Breast Cancer

**Figure 1:**
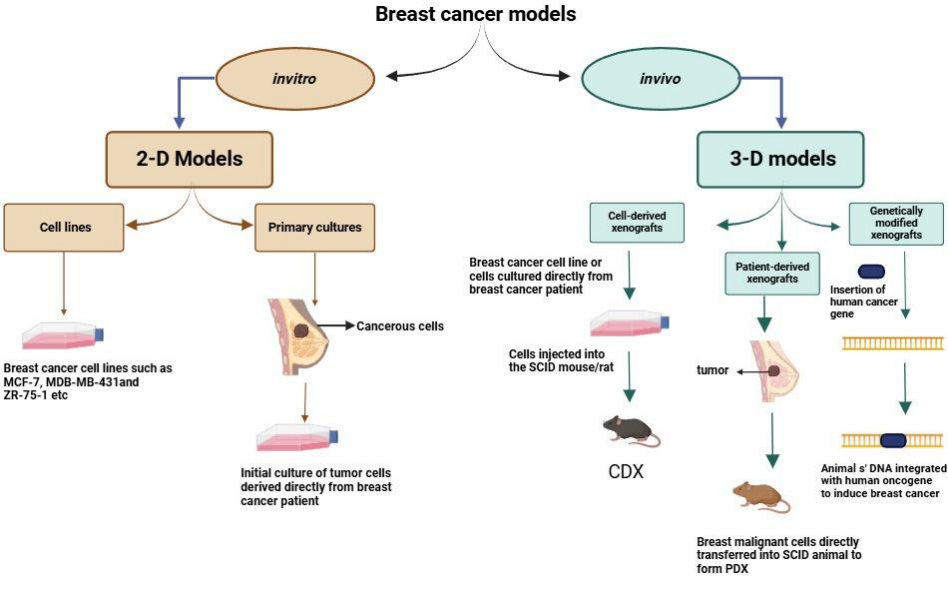
Graphical abstract: Pre-clinical models of breast cancer. Cell lines and primary cultures are two-dimensional models used *in vitro* while three-dimensional models such as cell-derived xenografts, patient-derived xenografts and genetically modified xenografts are utilized *in vivo *for breast cancer studies.

Preclinical models provide valuable insights into the mechanisms of drug metabolism, tumor genesis, and behavior. *In vitro* models serve as essential tools for the initial evaluation of drugs and novel compounds, facilitating a deeper understanding of the molecular mechanisms that drive drug action and contribute to drug resistance (Figure 1(Fig. 1), graphical abstract). The *in vivo *models provide a dynamic environment involving the immune system, vasculature, and other naturally occurring events in the (TME) tumor microenvironment such as cell-derived xenografts (CDX) and patient-derived xenografts (PDX). 

### Mimicking breast cancer in in vitro conditions

Mimicking breast cancer under *in vitro* conditions, that is replicating breast cancer cells outside the living body under controlled environment is one of the classical tools of breast cancer research which keeps advancing with new developments in the field. *In vitro* studies play a pivotal role in breast cancer research and enable the understanding of different cellular mechanisms in breast cancer as well as provide preliminary insights about drug response, efficacy as well as drug resistance. Established breast cancer cell lines or primary cultures isolated from mammary tumor biopsy are grown *in vitro* in 2D and 3D environments.

#### Breast cancer-derived cell lines (BCDC)

The first breast cancer cell line was developed by Lasfargues and Ozzello in 1958 from mammary tumor extracted from 74-year old breast cancer patient followed by the development of MCF-7 by Michigan cancer foundation providing a most commonly used *in vitro* model system for breast cancer studies (Holliday and Speirs, 2011[26]). Breast cancer modeling, which includes a panel of diseases with different phenotypical connections, has made extensive use of breast cancer cell lines (Table 1(Tab. 1); References in Table 1: Balcer-Kubiczek et al., 1995[3]; Bartek et al., 1990[4]; Chavez et al., 2010[11]; Christgen and Lehmann, 2007[12]; Davison et al., 2011[15]; Ding et al., 2021[18]; Engel et al., 2019[19]; Ferguson et al., 1997[20]; Hollestelle et al., 2007[24], 2010[25]; Leroy et al., 2014[34]; Mäki-Jouppila et al., 2018[39]; Pilco-Ferreto and Calaf, 2016[57]; Schafer et al., 2000[62]; Sweeney et al., 2018[64]; Vickers et al., 1988[71]; Vranic et al., 2011[73]; Welsh, 2013[75]). There are multiple breast cancer cell lines depending upon their origin, nature of breast cancer type they portray which as categorized as follows;

#### a) ER/PR positive breast cancer cell lines

Many cell lines with various molecular and phenotypic properties have been employed in breast cancer research. The luminal A condition (ER+, PR+/-, and HER2-), a subtype of breast cancer, is represented by the MCF-7, T47D, and SUM185 cell lines, whereas the luminal B condition (ER+, PR+/-, and HER2+) is demonstrated by the BTB74 and ZR-75 cell lines. Several essential features unique to the mammary epithelium are still present in the MCF-7 and T47D cell lines. They are known as ER-positive luminal A cell lines because they are hormone-dependent and responsive to estrogen through the cytosolic membrane's estrogen receptor alpha (ER) (Yu et al., 2017[79]).

The most researched is the MCF-7 cell line isolated from a pleural effusion in a breast cancer patient; a popular breast cancer cell line that has been propagated over many years by several researchers. It belongs to the luminal A molecular subtype and is estrogen and progesterone receptor (PR) positive. MCF-7 is a cell line that is generally thought to have a minimal propensity for metastasis since it is not aggressive and does not invade (Comşa et al., 2015[13]). Because these cell types are tumorigenic when exposed to estrogen, MCF-7 and T47D cell lines are widely employed to create xenograft tumor animal models. Although MCF-7 and T47D cell lines' phenotypic and molecular properties are known to be comparable, various investigations have found differences between the cell lines. According to investigations using mass spectrometry and two-dimensional gel electrophoresis, 164 proteins, for instance, are expressed differently (Yu et al., 2017[79]). 

However, there are certain disadvantages to MCF-7 cells. Genetic diversity and replicability are known to be significantly lacking in MCF-7 cells (Tran et al., 2021[69]). The MCF-7 cell line also exhibits the estrogen receptor mutations. However, it is unknown how this would affect responses mediated by estrogen receptors (Tora et al., 1989[68]). As Villalobos et al. (1995[72]) conducted a thorough investigation into the varying reactions of cells from various sources and found that cells from four origins responded to estradiol and nonylphenol in distinct ways. Further, TGF alpha is one of many non-estrogenic substances that can stimulate cell growth (Zacharewski, 1997[80]). Despite the fact that T47D and MCF-7 cell lines are luminal the data indicates that the cell lines represent a subtype of breast cancer and that the T47D cell line is a perfect *in vitro* model for progesterone-responsive luminal breast cancer (Yu et al., 2017[79]).

#### b) Triple-negative breast cancer cell lines

TNBCs are characterized with the absence of three hormone receptors namely, progesterone, estrogen, and HER-2 TNBC accounts for 15-20 % of all breast cancer cases, and patients with it typically have a worse prognosis than those with the other breast cancer subtypes (Obidiro et al., 2023[50]). In the majority of literature, cell lines with ER, PR, and HER-2 triple negative status are distinguished as basal A and basal B cell lines, with basal A being more luminal-like and basal B being more basal-like. While basal B cell lines tend to be more aggressive and exhibit mesenchymal and stem/progenitor-like qualities, basal A cell lines possess epithelial properties and are linked to BRCA1 gene profiles (Dai et al., 2017[14]).

Moreover, Triple-Negative A (basal A) lines are rich with basal markers such as integrins (ITGA6, ITGB4/6), cytokeratin (KRT4/5/6A/6B/13/14/15/16/17), LAMC2, LAMB3, S100A2, TRIM29, ANXA8, SLPI, BNC1, COL17A1, MET, CD10/14/58/59, LYN, thus are known as basal-like tumor and resemble the core basal tumor subtype. Triple-negative B (basal B) lines, designated the mesenchymal cluster or normal-like/claudin-low, over-express genes associated with tumor invasive and aggressive features such as COL3A1, COL6A1/2/3, COL8A1, MMP2/14, TIMP1, VIM, MSN, PLAT, A, CTSC, PLAU, PLAUR, HAS2, PRG1 5, 7, 8, SERPINE1/2, SPARC, FN1, FBN1, and cancer stemness such as CD44(+) and CD24(-) (Charafe-Jauffret et al., 2006[10]).

The MDA-MB-231 cell line is commonly utilized in models for late-stage breast cancer. This cell line is ER, PR, and E-cadherin negative and expresses mutated p53.The MDA-MB-231 cell genome clusters with the basal subtype of breast cancer in microarray profiling. The cells serve as a suitable model for triple-negative breast cancer since they are deficient in the growth factor receptor HER2. *In vitro* invasive MDA-MB-231 cells produce xenografts that spontaneously metastasize to lymph nodes when transplanted orthotopically (Welsh, 2013[75]).

#### Primary cultures and cell lines

The malignant cells obtained freshly from recent surgically removed tissue samples are grown in a complex media for a short period, known as primary cancer cell cultures (Nishikata et al., 2013[48]). These cultures preserve the original nature of the disease by providing a tumor microenvironment. The aim is to observe the interaction between malignant and healthy components *in vitro* (Miserocchi et al., 2017[44]). It is believed that cancer stem cells, or CSCs, are crucial targets for cancer treatment (Nishikata et al., 2013[48]), as the tumorigenic cells directly from the patient can help in better understanding of heterogeneity also initiation, development, advancement of metastasis of a tumor and may help in designing a personalized drug or therapy. The stem-like characteristic of cancer cells is maintained in primary cultures, which is beneficial for preclinical research on drug resistance pathways. Unlike the most popular immortalized cell lines, which might not entirely predict cancer expression, *ex vivo* models allow more precise representation of tumors and are better suited for clinical study (Miserocchi et al., 2017[44]).

The utilization of cell lines and primary cells as model systems presents numerous advantages, including ease of handling and an unlimited capacity for self-replication. Furthermore, cell lines exhibit a significant degree of homogeneity and can be promptly revived from frozen stocks in the event of contamination-related loss. However, there are certain drawbacks; during ongoing culture, cell lines are susceptible to changes in their genotype and phenotype. This phenomenon is particularly common in cell lines that are frequently utilized, especially those that have been preserved in cell banks for extended periods. Subpopulations may emerge due to the selection of specific, rapidly proliferating clones within a population, resulting in phenotypic changes over time that indicate the utility of *in vivo* model systems (Burdall et al., 2003[8]).

#### Normal breast epithelial cell culture as a negative control

Human mammary epithelial cells (HMECs) derived from disease-free breast tissue offer a unique and instructive system for investigating early stages of breast carcinogenesis (Wang et al., 1997[74]; Keller et al., 2010[29]). Different stages of breast carcinogenesis induced by different chemical, physical or biological carcinogens can be studied as these cells are normal and can be immortalized by transformation. HMECs can be used as negative control to compare the expression changes or effect of drugs on normal vs cancer cells. Further, HMECs are a heterogenous population consisting of four distinct epithelial states on the basis of differentiation with two luminal and two basal phenotypes. These states vary according to the expression of some of the marker proteins including CD24, EpCAM, and CD49f. These four cellular states are also present in primary breast cancer cultures, however, in different proportions as compared to healthy tissues. Additionally, uncommon basal and mesenchymal epithelial phenotypes, which are often seen in trace amounts in human tissues, are enriched in cultured cancer cell lines, offering an additional parameter which can be checked in breast cancer studies (Dewi and Cline, 2021[17]). However, none of these cells individually represent the complete breast cancer profile suggesting use of study design with multiple cell lines/types together for better understanding of the mechanism and drug effect. Some of the commercially available mammary epithelial cell lines include MCF10A, MCF10F, MCF12A, and MCF12F (Keller et al., 2010[29]; Dewi and Cline, 2021[17]).

### Mimicking breast cancer in in vivo conditions

The evolution and progress of basic and translational breast cancer research in humans have been profoundly impacted by animal models due to their high degree of resemblance to humans. The most frequently used animals for breast cancer research are rodents, particularly mice and rats, however, other primates like cynomolgus macaques (*Macaca fascicularis*), rhesus macaques (*Macaca mulatta*), and baboons (*Papio sp*.). More recently, New World Monkeys such as common marmosets (*Callithrix jacchus*) have also been utilized in mammary cancer-related studies (Dewi and Cline, 2021[17]). Mice are the most often used mammalian species because of their small size, low cost, quick generation time, and established gene editing technology (Jonkers and Derksen, 2007[27]). Additionally, there are numerous inbred mouse strains in the market. Understanding the molecular mechanisms behind mammary carcinogenesis is essential for the development of mouse models of breast cancer. By researching the dynamics and crucial elements of mammary gland development, the understanding of the progression of breast tumors has been significantly increased (Zeng et al., 2020[81]). Another important mammal model is the rat. Mceuen first noted the formation of mammary tumors (mammary fibroadenoma) in female rats in 1938, following the daily vaginal administration of an estrone solution in maize oil for two and a half years. The female rat has been utilized as a mammary cancer model ever since, and it is currently one of the most widely used animal models to investigate mammary carcinogenesis. 

The histological, biochemical, molecular, and genetic attributes of mammary carcinoma in female rats closely resemble those of human breast cancer. Moreover, rats yield a greater quantity of blood and tissue samples for subsequent studies compared to mice. Longitudinal studies have shown that specific rat strains can autonomously form mammary tumors, whereas those subjected to chemical carcinogens may exhibit accelerated tumorigenesis post-exposure. 

The tumor can be induced in different ways such as by using active tumor-inducing strains, chemicals and hormones. The design of research techniques heavily relies on the utilization of rat models that spontaneously produce breast tumors. Although the pituitary gland is the organ most usually impacted by the occurrence of spontaneous neoplasms in rats, the rat breast gland is the second most frequently affected organ, and it develops primarily following its first year of life (Alvarado et al., 2017[1]). Different types of rodent models include chemically induced, transplantable and transgenic breast cancer *in vivo* models.

#### Chemically induced mammary tumor 

The chemically induced cancers include certain synthetic chemicals which are carcinogenic in nature. For instance, 2-Amino-1-methyl-6-phenylimidazo[4,5-b]pyridine, 7,12-Dimethylbenzanthracene, N-Methyl-N-nitrosurea and so on (Liu et al., 2015[36]). DMBA, one of the most powerful polycyclic aromatic hydrocarbons is well known for its ability to cause mammary adenocarcinomas in both rats and mice. Many studies have been conducted on the molecular features of DMBA-induced breast cancer. The cancer induced by DMBA in female Sprague-Dawley rats was observed to be estrogen and progesterone-positive (ER/PR+) (Naruse et al., 2021[46]). In a study, mammary tumors produced by DMBA show high-frequency mutations in Hras and activate MAPK signaling. A missense switch from CAA to CTA transversion (Gln to Leu) at exon 2, codon 61 of the Hras gene was discovered by direct DNA sequencing. It was also demonstrated that DMBA induces tumors that are produced from both luminal and myoepithelial cells (Machida and Imai, 2021[38]). Because of its degree of similarity over other models, the model of chemically induced mammary tumors in female rats is still in use today. The tumors are easily induced, and the tumors' histology, hormone dependency, expression of estrogen receptors, and genetic alterations are similar to those found in humans. However, not all strains respond to all carcinogen agents well, some species are entirely resistant to these chemicals. For example, Sprague-Dawley easily develops mammary tumors, however, strain Copenhagen is resistant to DMBA-induced mammary carcinogenesis which could be due to genetics. Similarly, the chemical DMBA may not always be utilized to induce and evaluate the mammary tumor in rat models, however, certain chemicals such as bisphenol A, copper, resveratrol, folic acid, iron, Methylamoorain, milk, estrone sulfate, Phenethylisothiocyanate are found to increase breast neoplasm (Alvarado et al., 2017[1]).

#### Transplantable mammary tumors 

Transplantable models involve the implantation of cell lines either derived from established cell lines or directly from patients through surgery in animals. The cell line transplantation is known as cell-derived xenografts (CDXs). Similarly, the cells directly from the patient are transferred in animals termed as patient-derived xenografts (PDXs).

#### a) Cell-Derived Xenografts

**Figure 2:**
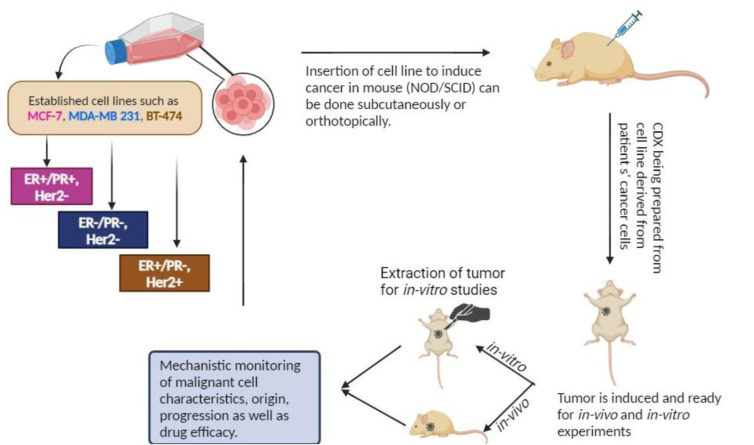
Cell-Derived Xenografts (CDX) Preclinical Disease Model of Breast Cancer. The cancer cells are cultured and passaged over time which is considered as an established cell line. The cell line is implanted into severe combined immunodeficient mice to record different parameters of BC carefully. The parameters involve initiation, progression, heterogeneity, etc.

CDX is one of the most basic and widely used model systems because it includes the engraftment of human cell lines into immunocompromised animals (Neve et al., 2006[47]). The investigation of malignant and metastatic characteristics in mice can be done using orthotopic CDX transplantation models, in which tumor cells are implanted into the mammary fat pad of NOD/SCID mice as shown in Figure 2(Fig. 2). Metastatic CDX transplantation models using cancer cells such as MDA-MB-231 and SUM149 cells introduced into the tail veins of mice are useful for observing experimental metastasis. These CDX models enable target genes of interest to be validated and facilitate the assessment of potential anti-cancer medicine and therapies for breast cancer (Park et al., 2018[53]). These have been demonstrated to be extremely useful for the assessment of breast cancer genetics, biological processes, and to some extent, metastatic potential. Additionally, the lines that are commonly employed are usually generated from extremely aggressive malignant tumors or plural effusions (fluids drained from lung metastases), such as the well-researched MDA-MB-231 line, making them less suitable for modeling early events in the progression of the source tumor. Well-characterized cell lines representing the common clinical subtypes - luminal A (e.g. MCF-7, T47D), luminal B (e.g. BT474, MDA-MB-361), HER2^+^ (e.g. SKBR3, HCC202) and triple negative (e.g. BT20, MDA-MB-231, MDA-MB-468) - have been extensively studied, not each can be evaluated in vivo (Holliday and Speirs, 2011[26]). However, they are constrained by their low intra-tumor heterogeneity and poor track record of predicting clinically effective treatments (Whittle et al., 2015[76]). 

**4T1 mammary carcinoma cell line: **Originally discovered by Fred Miller and colleagues in the early 1980s, the 4T1 mammary carcinoma is a transplantable tumor cell line. Both in tissue culture and on BALB/c mice, the tumor multiplies effectively. It is one of four sublines descended from the tumor, which was isolated from a single spontaneously occurring mammary tumor in a foster nursed MMTV+ BALB/c mouse that was being cared for by a C3H mother (Pulaski and Ostrand-Rosenberg, 2001[59]). To research more about the tumour metastatic heterogeneity with the objective of creating a 4T1 multi-organ metastatic imageable model (Paschall and Liu, 2016[54]), the experiment by Zhang et al. (2015[82]) involved the use of the right second mammary fat pad of BALB/c mice, which was an orthotopically injected stable 4T1 clone with strongly expressed Red Fluorescent Protein (RFP). Fluorescence imaging allowed for the easy visualization of the metastases. Extensive metastasis was seen in the lymphatic system and chest cavity through fluorescence investigation. The majority of the pulmonary parenchyma in all lobes was affected by large metastatic nodules in the lung. Fluorescent macroscopic metastatic nodules were seen in the liver beneath the capsule. Most frequently, the spine and thigh bone were affected by bone metastases (Zhang et al., 2015[82]). Removing the initial tumor appears to make it easier for metastasis to occur. These models are the breast cancer models with spontaneous metastasis that resemble patients with actual breast cancer and are useful for two things: researching spontaneous breast cancer metastasis pattern (4T1 cells, for instance, can be transfected with a gene of interest to test the functions of these genes in breast cancer spontaneous metastasis), and it should be helpful in the development and assessment of new therapies such as evaluating the effectiveness of chemotherapy and immunotherapy against spontaneous breast metastasis in an immune-competent host (Paschall and Liu, 2016[54]). The 4T1 syngeneic mouse tumor might be a suitable TNBC model for future research on angiogenesis and treatments as suggested by a recent study (Malekian et al., 2020[40]), which showed the lung metastasis of TNBC which was induced by injecting 4T1 in the right flank. The mRNA and protein expression of angiogenesis factors showed an increase in the early stages, which further increased dramatically in the final stage of tumor development. These tumors exhibited negative expression for ER, PR, and Her-2, thus validating the 4T1 mice model as an appropriate TNBC model for breast cancer. The 4T1 tumor has multiple features that render it an effective experimental animal model for breast cancer in humans including simplicity of transplantation into the mammary gland along with surety of developing the tumor in exact anatomic location, spontaneous tumor formation from 4T1 as this cell line is aggressive, metastatic potential to lymph nodes, bones and other organs as in case of human breast cancer. It is simple to remove the primary tumor surgically, allowing for the study of metastatic illness in an animal model that mimics the clinical scenario in which the primary tumor is physically removed but the metastatic foci are left intact. The fact that 4T1 is resistant to 6-thioguanine is another benefit. This characteristic makes it possible to accurately quantify metastatic cells, even when they are dispersed and at sub-microscopic levels in other organs. Furthermore, both *in vitro* and *in vivo* manipulation of the 4T1 tumor is rather simple (Pulaski and Ostrand-Rosenberg, 1998[60], 2001[59]).

This model also has some limitations including rapidly multiplying tumors that are frequently inoculated with many cells, creating a tumor microenvironment that does not accurately mimic human breast tumors, with unexpectedly low metastasis rate. When growth regression of primary tumors occurs (3-4 weeks) with substantial necrosis and leukocyte infiltration, it takes an extended period (5-6 weeks) for the parental 4T1 cells to generate metastatic tumors in distant organs in mice. Furthermore, after parental 4T1 cells were implanted in mammary fat pads (MFPs), the frequency of metastases was significantly reduced in mice. Thus, 4T1 cell-derived cell lines with improved metastatic capabilities are favored for studying the molecular roots of tumor proliferation (Guo et al., 2015[21]). Despite the broad application of the 4T1 animal model, the issues mentioned above seriously restrict its ability to shed light on the biology of metastatic breast cancer aid in the discovery of new treatment options and provide the necessary proof of concept.

#### b) Patient-derived xenografts (PDXs)

**Figure 3:**
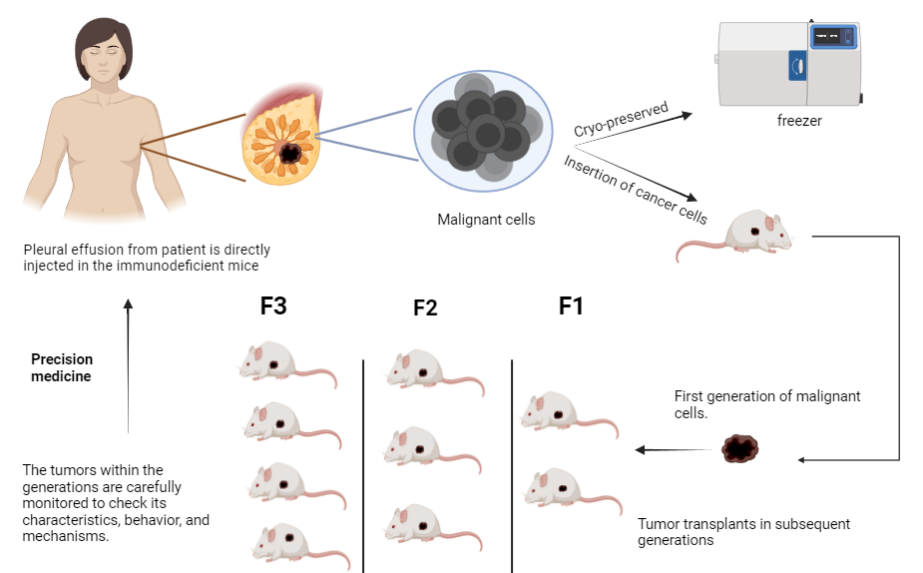
Patient-Derived Xenografts (PDX) Pre-Clinical Disease Model of Breast Cancer. The pleural effusion from the patient is directly transferred into rats or mice. The cells form a lump or tumor which is surgically excised by sacrificing the animal model. The tumor is transplanted to subsequent generations to observe the characteristics of breast cancer.

These are developed by implanting primary human cancer cells or tumor fragments into host mice, are considered preclinical models with enhanced therapeutic relevance and hold the capacity to overcome CDX-associated limitations. Although the transplantation of human tumor fragments into immunocompromised mice has a long history (Hoffman, 2015[23]), PDX models have recently attracted attention as the developed tumors preserve several key aspects of the primary human tumor, such as growth kinetics, histological features, behavioral traits (such as invasiveness and metastatic capacity), and most importantly, response to therapy (Marangoni et al., 2007[41]; DeRose et al., 2011[16]; Tentler et al., 2012[67]; Hidalgo et al., 2014[22]). The likelihood of engraftment is often higher the more immunocompromised the host is. The PDX line is regarded as "established" once the tumor tissue has been passed through three or more times (Figure 3(Fig. 3)) and a histological analysis has established that the PDX is a developing human tumor. The experiment then underwent morphological and genetic analysis for further disease alterations that occurred due to xenotransplantation (Marangoni et al., 2007[41]). Tumor engraftment was performed on female NOD/SCID mice that were five weeks old. Patients with breast cancer who had undergone surgical excision provided new tumor tissues, which were then inserted into the inguinal. After the tumor's portion was removed from the mouse, it was engrafted onto a second BALB/c nude mouse to grow. The aim was to observe the receptor alterations from the patient tumor to PDX. 

The IHC analysis found that the receptors ER, PR, and Her2 remained the same in both. The percentage of identity matching between the variations within 10 primary tumor PDX pairings ranged from 85 % to 96.9 %, while the percentage of identity matching between other PDXs and their primary tumors was only about 60 %. Furthermore, a remarkable degree of conservation between the main tumors and related PDX tumors were validated by clustering analysis of the variations. This confirmed that PDX and tumor share the same molecular signatures. Although TNBCs may be categorized into three classes, the RNA-seq data show that they are remarkably diverse. WB analysis of the important growth signaling molecules in TNBC PDXs revealed its diversity as well, which is consistent with this finding. This result supports the use of TNBC PDX as a representative model for the variety of TNBC tumors and is in line with earlier findings from primary TNBC tumor research (Jung et al., 2018[28]). The experiment by Park et al. (2019[52]) included the majority of the cancer tissue (65 among the 83 samples) from the 83 tumor samples obtained from TNBC patients. Mice were subcutaneously implanted with a single tumor sample. Mammary fat pads were injected with 78 tumors. 19 TNBC instances with successful engraftments of PDX models were identified. The mammary fat pads were the sites of all PDX model engraftments that were effective. The experiment by DeRose et al. (2011[16]) involved five tumor grafts that had ER+PR+ receptors, seven were ER−PR−, and five were HER2+. Eight grafts came from metastatic effusions, while four were from original tumors. The fat pads of female NOD-SCID (non-obese diabetic severe combined immunodeficiency) mice were used for the implantation of these tumors. Most of the tumor grafts here were metastatic and produced metastasis patterns resembling those observed in the original sufferers, although human lineages of breast cancer frequently have a low metastatic potential from the orthotopic location. It is significant to note that ER+ breast cancer models frequently experience loss of ER protein as a tumor grows or after several transplants. The histology, clinical indicators, gene expression profiles, variation in copy number, and estrogen dependency and/or responsiveness of the original tumors are all retained in these grafts. By boosting the vascularization of the tumors, the inclusion of MSCs promotes graft development and helps to sustain ER expression. The capacity of the breast tumours in the study of individuals and the comparable grafts to spread spontaneously is one of the most encouraging similarities.

In patients with primary TNBCs, PDX model success rates were higher than in cases with residual TNBCs after treating with neoadjuvant therapy, and a PDX model with the unique germline harmful BRCA1 mutation L1780P was developed. T-stage, histologic grade, estrogen receptor status, and Ki67 levels were statistically important in the univariate analysis. Death, aggressive disease status, or the presence of a BRCA mutation were all associated with effective PDX models. The findings were not notable for hormone and HER2-positive cases, which made up a small percentage of all cases. TNBC and Neo TNBC cases were used to construct all successful PDX models. In TNBC instances, tumor size was insignificant. Histologic grade, BRCA mutation presence, aggressive condition, and mortality were statistically significant in TNBC subjects treated with neoadjuvant chemotherapy. Compared to TNBC PDX models, neo-TNBC PDX models reported shorter establishment period intervals. It has been demonstrated that the aggressiveness of tumors has an impact on the development of PDX models as well as the quick development of tumors and tumor growth in immune-deficient mice. It agreed with our research. Therefore, residual TNBC following chemotherapy with neoadjuvant therapy may be more favorable with establishment periods for developing PDX models than the initial tumor of TNBC. In conclusion, for creating effective PDX models, patients with aggressive disease status, enlarged tumors, and poor histologic grade are ideal (Park et al., 2019[52]).

#### c) Genetically engineered mouse models (GEMMs)

**Figure 4:**
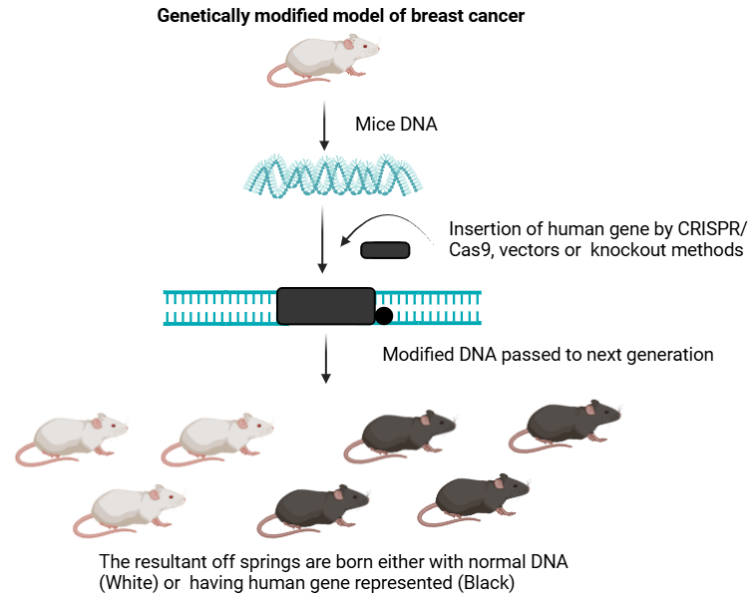
Genetically Modified Pre-Clinical Disease Model of Breast Cancer. The breast cancer-causing gene is integrated into the mouse DNA, which expresses and results in breast cancer. The type of breast cancer, a model exhibited depends on the type of BC gene inserted in its genome.

The genetically modified, transplantable mice models enable the investigation of breast tumor immunity. Following the current existing protocols, pharmacological therapies are administered after orthotopically transplanting breast tumor pieces into the mammary fat pad. These preclinical models offer useful methods for examining the biology of breast tumors, treatment responses, the identification of biomarkers, and drug resistance mechanisms. The study of spontaneous tumor development, progression, and metastasis in the context of an intact immune system is made possible by genetically engineered mice models (GEMM), such as Trp53 homozygous null, cMyc, Wnt1, PyMT, or Her2 overexpression models (Figure 4(Fig. 4)) (Lv et al., 2020[37]).

Transgenic models are prepared by the insertion of source DNA into a fertilized mouse egg which is then transferred to the foster female mouse. These models are particularly useful for studying the early stages of the tumor process. In cancer biology, the GEMMs are required to express the human oncogene by integrating it into the mouse DNA and creating a tumor having similar human tumor parameters such as heterogeneity, metastasis and active tumor sites. In these models, a mammary cell would normally initiate a spontaneous tumor when the proper microenvironment is present. These might be straightforward oncogenic transgenic mice, often known as standard GEMM (for example, MMTV-PyMT). Preclinical models created by gene editing technologies can mimic spontaneous mammary carcinogenesis by expressing one or more potential oncogenes, such as MYC, HRAS, and PIK3CA, under the transcriptional control of mammary-specific promoters. On the other hand, the Cre-Lox system permits targeted knock-in of potential oncogenes or tissue-specific deletion of tumor suppressors including p53, Rb1, and Brca1. 

In this way, genetically modified mice models (GEMMs) may mimic specific subtypes of breast cancer and are widely used to investigate the effects of genetic modification on the genesis, development, and metastatic spread of mammary tumors (Regua et al., 2021[61]).

The Polyoma Virus Middle T (PyMT) antigen breast carcinoma transgenic mouse model is widely used for simulating human breast cancer due to its high rate of pulmonary metastasis, rapid tumor development, and great penetrance. The tumor development of PyMT tumors is well-characterized, with tumor cells decreasing ER/PR expression and increasing HER2 levels when the tumor develops into mammary intraepithelial neoplasia (MIN) and late carcinoma (Lin et al., 2003[35]). Although these models may not accurately represent oncogenes in clinical tumors, they are still useful in breast cancer research. PyMT-derived tumors have been discovered to be useful for researching luminal B subtype tumors and triple-negative breast cancer, as they have an early loss of ER/PR expression. Advanced breast tumors often exhibit overexpression of receptors on cell surfaces, which have grown in favor of therapeutic targets for breast cancer (Pfefferle et al., 2013[56]).

Carboni et al. (2005[9]) created a transgenic mouse model of breast carcinoma that uses the MMTV promoter to drive the overexpression of the IGF-IR in the salivary and mammary glands, duplicating the overexpression of IGF-IR commonly observed in breast malignancies. Stewart et al. (1984[63]) created the first transgenic rat model for breast cancer, which is called MMTV-c-myc. Many cancer forms, including breast cancer, have been linked to cellular myc (c-myc). This strain's transgenic females develop localized aggressive mammary adenocarcinomas with elevated mitotic indices; however, their tumor duration is prolonged (200 days). Tumors derived from this model are projected to be of the basal and luminal B subtype, according to transcriptome analysis, which implies that c-Myc overexpression might be helpful in the *in vivo* research of both breast cancer subtypes (Pfefferle et al., 2013[56]). The biology and pathology of this illness can be studied using genetically modified mouse models of breast cancer, which have many characteristics of human breast cancer. As molecular profiling studies multiply, a framework for contrasting GEM and human breast cancer is provided. No single model can fully capture all facets of breast cancer due to its complexity and heterogeneity. Therefore, the most effective technique to represent this condition at this time is through an integrated and multi-systems approach (Vargo-Gogola and Rosen, 2007[70]).

The study of cancer has greatly benefited from the use of genetically modified mice (GEMMs). Unlike the malignant cell implantation models, GEMMs create tumors from scratch in a microenvironment that is naturally immune-sufficient. The tumors that develop in advanced GEMMs exhibit genetic variability, can move spontaneously toward metastatic illness, and closely resemble the histological and molecular characteristics of their human counterparts. As a result, GEMMs are often preferred to cancerous cell inoculation models, which exhibit little to no heterogeneity and are frequently metastatic from the beginning. These models are essential for preclinical research because they capture both tumor cell-intrinsic and cell external variables that contribute to de novo tumor genesis and development toward metastatic illness (Kersten et al., 2017[30]). Besides their immense merits, they also exhibit some critical demerits that affect the expected results. The fact that the genetics and histology of the tumors are usually not typical of the relevant human malignancies is another drawback of utilizing genetically modified models, and up until now, these models have not been as predictive of treatment effectiveness as one would hope (Becher and Holland, 2006[6]).

#### d) Non-human primates (NHPs)

NHPs are more comparable to humans in terms of their genetic evolution, anatomy, physiology, biochemistry, and organ systems when compared to rodents (Xia and Chen, 2011[78]). According to research by Puente et al. (2006[58]), nearly all human cancer genes are significantly conserved between chimps and humans. Three different types of monkeys lived in the colony: cynomolgus monkeys (*Macaca fascicularis*), rhesus monkeys (*Macaca mulatta*), and African green monkeys (*Cercopithecus aethiops*). Since the program's inception in 1961, all three species have been bred at the institution and have been the main supply of experimental animals (Takayama et al., 2008[65]). 

A study by The North Carolina team recently was able to characterize 35 mammary gland lesions in cynomolgus and rhesus macaques at various NIH-sponsored primate research centers in the United States, ranging from ductal hyperplasia to carcinoma in situ and invasive ductal carcinomas. The development of morphologically preneoplastic changes, upregulation of proliferation markers in mammary duct epithelia, alterations in estrogen and progesterone receptors, growth factor receptors, and markers of cell death are just a few of the events they have shown in this model that are similar to those described in human breast cancer also pinpointed the similarities of breast cancer among both species. For macaques, the gastrointestinal tract, hematological system, dermis and subcutis, and female reproductive tract are the most often involved areas of tumor development. Mammary tumors are uncommon, although according to published literature, the lifetime incidence of mammary gland carcinoma ranges from 4 to 8 %. Since it is still poorly understood, this illness is currently regarded as infrequent among nonhuman primates (Tarara, 2007[66]). In addition to monkeys, canines have more than 80 % of the genetic makeup similar to humans (Mottolese et al., 1994[45]). Upon examining the histological morphology and clinical features of 21 dogs with spontaneous inflammatory breast cancer, Peña et al. (2003[55]) concluded that the cancer can serve as a spontaneous model for human inflammatory breast cancer. The establishment of mammary gland neoplasia in nonhuman primates has no known viral etiology. Initially believed to be carcinogenic, Mason-Pfizer monkey virus (MPMV) was discovered in a mammary cancer of a rhesus monkey. However, further research has revealed that MPMV's main pathogenesis is immunosuppression. The diversity of tumors observed in monkeys in contrast to the reproducible specific tumor types produced in other animal models, such as chemically induced mammary gland adenocarcinomas of rats and oncogene-specific mammary gland tumors with relatively uniform morphology in genetically modified mice, has called into question the usefulness of the non-human primate model for human breast cancer. The comparatively low occurrence of malignant tumors in macaques also raises questions about the merits of the monkey model. The incidence of recorded tumors is about 5 % for all age categories, and the nature of neoplasia varies among the various nonhuman primate communities. The non-availability of such a population, long incubation periods and expensive procedures of maintaining the whole experiment adds additional demerits to the use of non-human primates as breast cancer models. However, science is looking forward to bringing this species as an effective and efficient model for breast cancer (Tarara, 2007[66]). 

## Conclusion

The progression of preclinical models, from conventional 2D cell cultures to advanced 3D models that more precisely resemble the tumor microenvironment, has significantly enhanced anti-breast cancer research. The advantages of 3D models, including their capacity to replicate tissue architecture and cell-cell interactions, hold significant promise for enhancing our comprehension of diseases and facilitating the development of more efficacious treatments. These constraints highlight the essential necessity of amalgamating preclinical model investigations with clinical research to facilitate a seamless transition from bench to bed side. Despite substantial progress, some unresolved questions persist in the field, including precise models of breast cancer subtypes, advanced techniques for assessing therapy success *in vitro*, and the development of authentic tumor microenvironments. By rectifying the deficiencies of both 2D and 3D models, we can ultimately enhance patient outcomes and advance breast cancer research. Future efforts must focus on multidisciplinary collaboration and the development of innovative methodologies for model generation and characterization to address these research gaps. The field of genetic engineering has the capacity to enhance models in accordance with the specified needs, addressing the aforementioned limitations. This may further assist in reducing costs and time by employing animals that are more adaptable and less resistant than the existing versions.

## Declaration

### Consent for publication

All authors have read the article and consented to publish the manuscript.

### Competing interests

The authors declare no competing interests.

### Authors' contributions

Ravneet Kaur and Anuradha Sharma perceived the concept and designed the structure of the review. Ravneet Kaur wrote the first draft. Anuradha Sharma and Nalaka Wijekoon critically reviewed the manuscript and constructed the final draft. The figures were perceived by Anuradha Sharma and created by Ravneet Kaur.

### Acknowledgments

AS and RK are thankful to Lovely Professional University for providing the environment in which we conducted this review.

## Figures and Tables

**Table 1:**
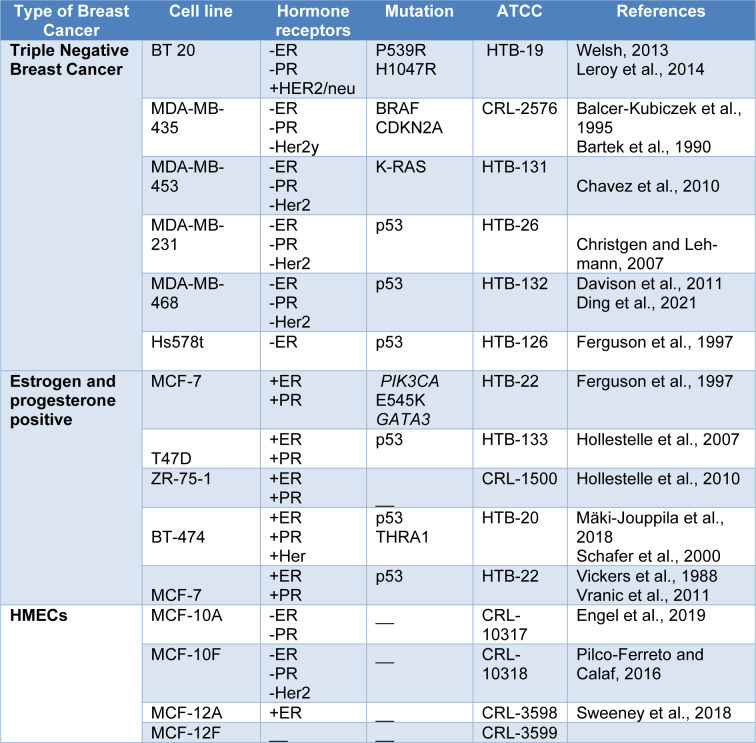
Cell lines as preclinical breast cancer models: The different cell lines with their altered hormone status, the type of mutation occurring in a particular breast cancer type and ATCC number.
